# The COVID-19 Family Stressor Scale: Validation and Measurement Invariance in Female and Male Caregivers

**DOI:** 10.3389/fpsyt.2021.669106

**Published:** 2021-05-28

**Authors:** Heather Prime, Mark Wade, Shealyn S. May, Jennifer M. Jenkins, Dillon T. Browne

**Affiliations:** ^1^Department of Psychology, York University, Toronto, ON, Canada; ^2^Applied Psychology and Human Development, University of Toronto, Toronto, ON, Canada; ^3^Department of Psychology, University of Waterloo, Waterloo, ON, Canada

**Keywords:** COVID-19, family stress, caregivers, child mental health, scale validation, measurement invariance

## Abstract

The COVID-19 pandemic has raised significant concerns regarding the effect of social disruptions on parental mental health, family well-being, and children's adjustment. Due to the pace of the pandemic, measures of pandemic-related disruption have not been subject to rigorous empirical validation. To address this gap, a multi-national sample (United Kingdom, 76%; United States, 19%; Canada, 4%, and Australia, 1%) of 372 female caregivers and 158 male caregivers of 5–18-year-old children was recruited online. Participants completed a survey including a 25-item scale indexing disruption in finances, basic needs, personal and family welfare, career/education, household responsibilities, and family relationships related to the pandemic. An exploratory factor analysis yielded an optimal three-factor solution: factors included Income Stress (five items related to income, debt, and job loss; loadings ranged from 0.57 to 0.91), Family Stress (seven items related to family altercations and child management; loadings from 0.57 to 0.87), and Chaos Stress (four items related to access to supplies, crowded shopping areas, news coverage; loadings from 0.53 to 0.70). Multiple-group confirmatory factor analysis demonstrated measurement invariance of each factor across female and male caregivers, indicating that factor structure, loadings, and thresholds were equivalent across groups. Composites reflective of each factor were computed, and Mann-Whitney *U* tests indicated that female caregivers consistently scored higher than male caregivers on COVID-19 stressors related to income, family, and chaos. Finally, concurrent validity was demonstrated by significant bivariate correlations between each scale and caregiver, family, and child outcomes, respectively. This demonstrates the validity of the COVID-19 Family Stressor Scale for use with female and male caregivers in family-based research. The current sample was predominantly White-European, married/common-law, and had at least some post-secondary education. Additional sampling and validation efforts are required across diverse ethnic/racial and socioeconomic groups.

## Introduction

The COVID-19 pandemic has brought upheaval to families across the globe. There is widespread disruption to family life due to school closures, loss of access to regular childcare, social distancing, household crowding, economic recession and its associated consequences (e.g., job loss, loss of employer-sponsored insurance, and food insecurity), and disruptions related to managing the pandemic such as (perceived or actual) shortages of supplies and an influx of news/media coverage (1, 2). Such social disruptions pose a significant threat to the mental health of parents and children, in part due to the potential for adverse changes to family systems and relationships with reciprocal and self-maintaining effects (3, 4). Indeed, mental health symptomatology in children and parents is dramatically elevated compared to pre-pandemic estimates, with the emergence of stress-related disorders and the exacerbation of pre-existing mental health difficulties (5–7). It is thus critical to assess the nature of social disruptions in families that are emanating from the COVID-19 pandemic. Such an endeavor will aid researchers, practitioners, and policy makers in child and family health services to understand the extent and nature of the social consequences of COVID-19 to families.

To date, there does not exist an empirically-sound and comprehensive measure of family stressors related to COVID-19. Though COVID-specific psychological distress and mental health scales for use with adults have been validated (8), there is no validated scale to assess for the range of social disruptions due to the pandemic, nor is there a scale that is tailored to the needs of families with children in the home. Some family studies include single COVID-specific items (e.g., the percentage of participants who have applied for a federal relief benefit; have had a reduction in available childcare; or have experienced job loss due to the pandemic) (2, 7, 9). Others have developed scales for use as a checklist, with items indexing pandemic-related stress resulting from new work and parenting demands (10), stressors related to stay-at-home restrictions and school/childcare closures (11), and/or a combination of challenges (e.g., family altercations, work/school demands, concerns about the health, pandemic-related news) and sources of resilience (e.g., family time) (12, 13). Despite this important work, there does not yet exist a comprehensive scale for family-related stressors during COVID-19 with demonstrated psychometric validity.

Another problem related to family research during COVID-19 is the pattern—well-documented in developmental psychopathology—of omitting male caregivers from observational or survey-based studies (14). Inclusion of both male and female caregivers in research examining the adverse impact of social disruptions due to COVID-19 is essential given the apparent disproportionate impact of the pandemic on female caregivers (15, 16). For instance, young women are at particular risk for moving out of the workforce during the pandemic, possibly due to the increase in childcare responsibilities (17). Furthermore, mothers have reported increased levels of psychological distress, anxiety, and depression compared to pre-pandemic levels (7, 18, 19). Further investigation into the disparate impact of social disruptions related to COVID-19 on male and female caregivers, and the implications this has for family well-being and child adjustment, is warranted. As such, any measure of stressors to families during COVID-19 needs to consider conceptual and measurement issues related to differences in female and male caregivers during this time. This difference pertains to the structure and organization of stressors (i.e., whether stressors cluster together in similar ways to capture meaningful dimensions of COVID-19 disruption) as well as the level of disruption experienced by male vs. female caregivers.

### Current Study

The aim of the current study is to develop and validate a measure of COVID-19-related psychosocial stressors to be utilized in family-based research—the COVID-19 Family Stressor Scale (CoFaSS). The CoFaSS was developed within a conceptual framework of COVID-19 disruption and family resilience, further described below (20). We follow steps for the development of a multiple-item scale, outlined by Warner (21), including generating the item pool, administering the questionnaire to a large group of participants, factor analysis of responses, scale formation, and an assessment of scale reliability and validity. In line with the *Standards for Educational and Psychological Testing* (22), the current paper conceptualizes validity as a unitary concept, referred to as construct validity (23). We utilize various sources of validity evidence to support the construct validity of the CoFaSS. First, we examine the internal structure of the scale. Specific attention is paid to measurement invariance across caregiver sex to ensure that the scale has similar structure and meaning to male and female caregivers, a requirement prior to using the scale to compare groups or/or in predicting other constructs that are expected to vary as a function of increased stress (e.g., mental health) (24).

Next, we examine the resultant CoFaSS scales for internal consistency and their relations to other variables (i.e., concurrent validity). With respect to the latter, we expect there to be mean group differences across male and female caregivers in social disruptions related to the pandemic, as captured using the CoFaSS scales. Concurrent validity is further examined within a theoretical model linking COVID-19 to child and family well-being (20). Specifically, social disruptions resulting from the COVID-19 pandemic are hypothesized to adversely affect family relationships through their impact on caregiver well-being. These negative changes to the family unit are, in turn, likely to have a cascading effect on children's well-being across several domains. In line with this conceptual framework, CoFaSS scores were expected to relate to theoretically-relevant caregiver outcomes (indexed by mental health and parenting stress), family outcomes (indexed by couple satisfaction, marital conflict, and parenting practices), and child outcomes (indexed by anxiety, depression, and anger). Associations were expected to be in the small to moderate range given the multiple determinants of complex human processes such as family relationships and mental health (25, 26).

This endeavor addresses current measurement limitations, such as the use of non-validated scales and/or single-item metrics that do not adequately capture the variegated and cumulative ways in which the pandemic has disrupted life for families. The goal of this project is to inform how pandemic adversity is conceptualized, measured, and studied, while providing a practical tool that can be easily and reliably deployed in child, youth, and family research during times of international crisis.

## Methods

### Procedure

Data come from the first wave of data collection of the *Child Resilience and Managing Pandemic Emotional Distress in Families Study* (CRAMPED), a multi-national, longitudinal study examining family dynamics and sibling differences during COVID-19. Ethics approval for the current study was obtained from the research ethics boards of the universities of all listed authors. Recruitment for the larger study was conducted from an online research panel (Prolific®). Prolific® is a research company that facilitates online participant recruitment for surveys, including the targeting of specific populations, such as parents/caregivers, as is the case in the current study. This occurs through screening an ongoing pool of over 70,000 panelists worldwide. Based on availability of financial resources and statistical power, a target sample of 1,000 children in 500 families was established for the current study. The survey was launched in May 2020 and made available for approximately 1 week. Panel members from Prolific were initially screened based on a question determining if they had two children between 5 and 18 years given the study's goal of studying within-family processes in developmental psychopathology. Panel members who were eligible were invited to complete the study survey on Qualtrics®. There were 3,200 panelists screened, 626 who met inclusion criteria, and 549 who completed the survey within the time period that the survey was active. Panel members were remunerated based on the amount of time it took them to complete the survey (i.e., the survey's length). The survey for data included in the first wave of the study took approximately 56 min to complete. The average payout for the survey completion was $10.80 USD/participant. All questions were completed by a single caregiver, including questions on caregiver and child demographics, COVID-19 stressors, disruptions, and potential benefits, caregiver mental health and childhood experiences, family relationships and functioning, and child adjustment (for two children).

### Participants

Participants included 549 caregivers (age: *M* = 41.33, *SD* = 6.329), who reported on themselves and their children (*N* = 1,098; younger child *M*_*age*_ = 9.62, *SD*_*age*_ = 3.21, 45.9% female; older child: *M*_*age*_ = 11.80, *SD*_*age*_ = 3.32, 49.0% female). Caregivers were mostly female (68%), married/common-law (90%), White-European (73%), and had at least some post-secondary education (69 %). The majority of the respondents resided in the United Kingdom (76%), with others residing in the United States (19%), Canada (4%), and Australia (1%). There was a wide range in annual household income prior to the pandemic (<$15,000 to $175,000+ USD), with the median value falling in the $50,000 to $75,000 USD range. Data on caregiver sex was extracted from the Prolific® database, with options of “male,” “female,” or “prefer not to answer.” Of the original sample, 530 caregivers elected to report on their sex (372 female and 158 male). This subset of participants comprised the final sample for the current study. The current study only reports on measures involving the younger child.

### Measures

#### COVID-19 Family Stressor Scale—Item Pool

The 25-item pool was generated by the principal investigator of the CRAMPED study (DB) and members of their laboratory at the University of Waterloo, Canada, and was subsequently reviewed with critical feedback from the research team (authors of the current paper, HP, JJ, MW, SM). The conceptual framework for the items was based upon a theoretical model of COVID-19 disruption and family resilience, which draws from systemic models of human development and family functioning, as well as empirical findings on the negative consequences of cumulative risk, human-made and natural disasters, global health crises, and economic recessions (20). In particular, the research team considered various stressors emanating from the pandemic and read available scientific and popular media reports that were emerging in the initial days and weeks of the pandemic (March 2020). A convenience review of existing disaster literature was conducted (27). Websites of the World Health Organization and Center for Disease Control were also reviewed to glean early insight into the nature of the unfolding disaster. The consensus was that 25 items exhaustively covered the content area of pandemic disruption for families. Items were revised for readability, and a final Flesch-Kincaid Readability analysis of 9.6 was deemed acceptable for the current purposes.

Items included stressors across domains of finances, basic needs, personal and family welfare, career and education, and household responsibilities. A list of all original items can be seen in Table 1. Participants were asked “Since the COVID-19 disruption, have any of the following changes occurred in your household?” and reported the level of applicability for each type of stress on a three-point Likert scale [“*Not True*” (1), “*Somewhat True*” (2), and “*Very True*” (3)]. A “not applicable” option was not available for respondents. Items were scored based on respondent endorsements and there was very minimal missing data. Thus, for those items that were not applicable for participants, the default response was likely (1) (i.e., not true). The internal structure, psychometric properties, and concurrent validity of the resulting subscales are presented in the Results.

**Table 1:** Original scale items.

**Item #**	**Item description**	**Subscale^a^**
1	Significant decrease (over 10%) in household income	Income
2	Gone into financial debt	Income
3	Unable to pay rent or mortgage	–
4	Job disruption or loss (myself or my partner)	Income
5	Could not access essential supplies (e.g., sanitizer, soap, toilet paper, etc.)	Chaos
6	Overwhelmed by the amount of COVID-19 news coverage	Chaos
7	Applied for employment insurance or government assistance	Income
8	Became concerned about providing for family	Income
9	Became stressed by crowded grocery stores and shopping centers	Chaos
10	Lost substantial money in the stock market (over 10% of holdings)	–
11	Working from home while meeting family demands	–
12	Closed a business or laid off employees	–
13	Experienced increased altercations with family members	Family
14	Experienced increased emotional withdrawal from family members	Family
15	Children have become harder to manage	Family
16	Inability to access educational materials for children	Family
17	More relationship conflicts with my partner (if I am in a relationship)	Family
18	Struggled emotionally with the loss of routine	–
19	Difficulty developing a new family and/or personal routine	Family
20	Felt crowded in my living space	Family
21	Significant anxiety/panic about danger to myself or loved ones	Chaos
22	Separated from family or loved ones due to COVID-19	–
23	Lost family or a loved one due to a COVID-19 related death	–
24	I have taken on increased responsibilities, beyond those of my family members.	–
25	Other disruptions not listed here	–

a *Items denoted ‘–’ were not included in final scales; all items included in a subscale were also included in the General Stress scale*.

#### Validation Scales

##### Caregiver Outcomes

*Caregiver anxiety*. The short-form, four-item, anxiety measure of the Patient-Reported Outcomes Measurement Information System [PROMIS® v1.0; (28)] measures frequency of feelings of fear, worries, and anxiety in the past 7 days, with responses ranging from “*Never”* (1) to “*Always”* (5). Internal consistency in the overall sample was very good (α = 0.92).

*Caregiver psychological distress*. The Kessler Psychological Distress Scale (K10) (29) is a widely utilized, 10-item scale assessing the frequency of feelings related to depression and anxiety as experienced in the past 30 days, with response options ranging from “*None of the time”* (1) to “*All of the time”* (5). Responses yield a global score of distress (α = 0.93).

*Parenting stress*. Parents were asked the follow question: “Over the past 14 days, how stressful were your parenting experiences with [child name]?” and asked to respond using a seven-point scale ranging from “*Not at all stressful*” (1) to “*Extremely stressful*” (7). This item has demonstrated validity (30).

##### Family Outcomes

*Caregiver-partner relationship satisfaction*. The brief Couples Satisfaction Index (31) includes four items related to happiness, comfort, and satisfaction within the couple relationship, using six to seven-point Likert scales (α = 0.94).

*Caregiver marital conflict*. Four items from the 2014 Ontario Child Health Study (32) were used to assess conflict between partners. Caregivers reported on the frequency of minor and major disagreements, in addition to the presence of minor and major physical aggression (e.g., pushing, shoving, or slapping, and punching, kicking, or beating). A composite score was created in which more frequent disagreement or aggression represented greater marital conflict (α = 0.57).

*Parenting practices*. Caregivers reported on their own parenting practices using the revised version of the Parenting Practices Scale from the 2014 Ontario Child Health Study (32). The caregiver reported on the frequency of five positive parenting practices (e.g., “I give [child] a lot of care and attention;” “I listen to [child's] ideas and opinions”) and five negative parenting practices (e.g., “I nag [child] about the little things;” “I say mean things to make [child] feel bad…”) in the past month on a five-point scale ranging from “*Never*” (1) to “*Always*” (5). A summed score was calculated (negative parenting practice items were reverse scored) and a higher score indicated greater positive parenting (α = 0.81).

##### Child Outcomes

Caregivers reported on children's mental health problems using the parent proxy reports of the Patient-Reported Outcomes Measurement Information System (PROMIS®). The following domains were administered: anger (v2.0, five-items) (33), anxiety (v2.0, eight-items) (34), and depressive symptoms (v2.0, six-items) (35). Caregivers reported the frequency of difficulties in each domain on a five-point Likert scale ranging from “*Never*” (1) to “*Almost Always*” (5), α > 0.85 across domains.

### Data Analysis

We used MPlus version 8.5 (2012–2020) to conduct the exploratory factor analysis (EFA) and tests of measurement invariance, and SPSS version 27 for descriptive statistics and analyses assessing concurrent validity. Minimal missing data were present (<1% for any variable).

#### Internal Structure

After removing low-frequency items, we subjected remaining items to an EFA (including 1–5 factors) in order to examine the underlying structure and interrelationships of scale items. This analysis used geomin rotation and the default weighted least squares estimator for categorical/ordinal indicators. We examined eigenvalues to identify potential factor solutions (based on eigenvalues > 1) and examined the empirical factor solution, in conjunction with conceptual accuracy, as the basis for grouping items into scales. Test of model fit relied on a non-significant chi-square value, as well as indices that are not sensitive to sample size including the comparative fit index (CFI ≥ 0.95) and the root mean square error of approximation (RMSEA ≤ 0.06) (36). We used a cut-off for geomin factor loadings of ≥0.50 in deciding which indicators to retain. Following selection of a final factor solution, we examined intercorrelations between factors.

#### Measurement Invariance

We examined measurement invariance across caregiver sex to compare and contrast the latent structure of the CoFaSS scale for male and female caregivers, independently for each factor. We used multiple-group confirmatory factor analysis (MGCFA), wherein two nested models of varying restriction—a constrained model and an unconstrained (baseline) model—were compared for significant differences in model fit. The grouping variable was caregiver sex. If the model fit of the constrained model was not significantly worsened as compared to the baseline model, then invariance of the tested parameters was accepted (37). Specific steps of MGCFA test for measurement invariance are outlined below.

First, we obtained the best factor model for each group separately by conducting independent CFAs. Mis-specified parameters were addressed following guidelines from Byrne (38). Once the model was fit to each group (male and female caregivers), the Mplus shortcut for measurement invariance was used to assess invariance across groups. Configural invariance was examined first, which assesses whether the same factor model is supported across groups, without any constraints, representing the baseline model. Metric and scalar invariance (i.e., whether the factor loadings and item thresholds, respectively, are equivalent across groups) were tested simultaneously by the addition of equality constraints on factor loadings and item thresholds across groups (hereafter referred to as the “scalar” model). The MPlus MI shortcut compares chi-squares for configural and scalar measurement models. A significant chi-square difference test indicates a significantly worsened model fit of the scalar as compared to the configural model. However, given the sensitivity of change in chi-square to sample size, change in CFI (<-0.01) and change in RMSEA (<0.01) were used as additional cut-offs for assessment of meaningful change in model fit (24, 39). Evidence of configural, metric, and scalar invariance are required to establish strong measurement invariance.

#### Scale Formation and Concurrent Validity

Following tests of measurement invariance, scales were formed by summing scores within each factor, in addition to a general stressor scale including all items. Internal consistency of each scale was assessed by obtaining a Cronbach's alpha. Intercorrelations among all scales were examined using Spearman's rho. Relations of the CoFaSS scales to other variables was used as a test of concurrent validity. We examined whether the CoFaSS scales captured mean rank differences across male and female caregivers using Mann-Whitney *U* Test (due to the non-parametric shape of the data). We also examined the bivariate correlations between CoFaSS scales and several concurrent caregiver, family, and child outcomes, using Spearman's rho.

## Results

### Internal Structure

**Figure 1:**
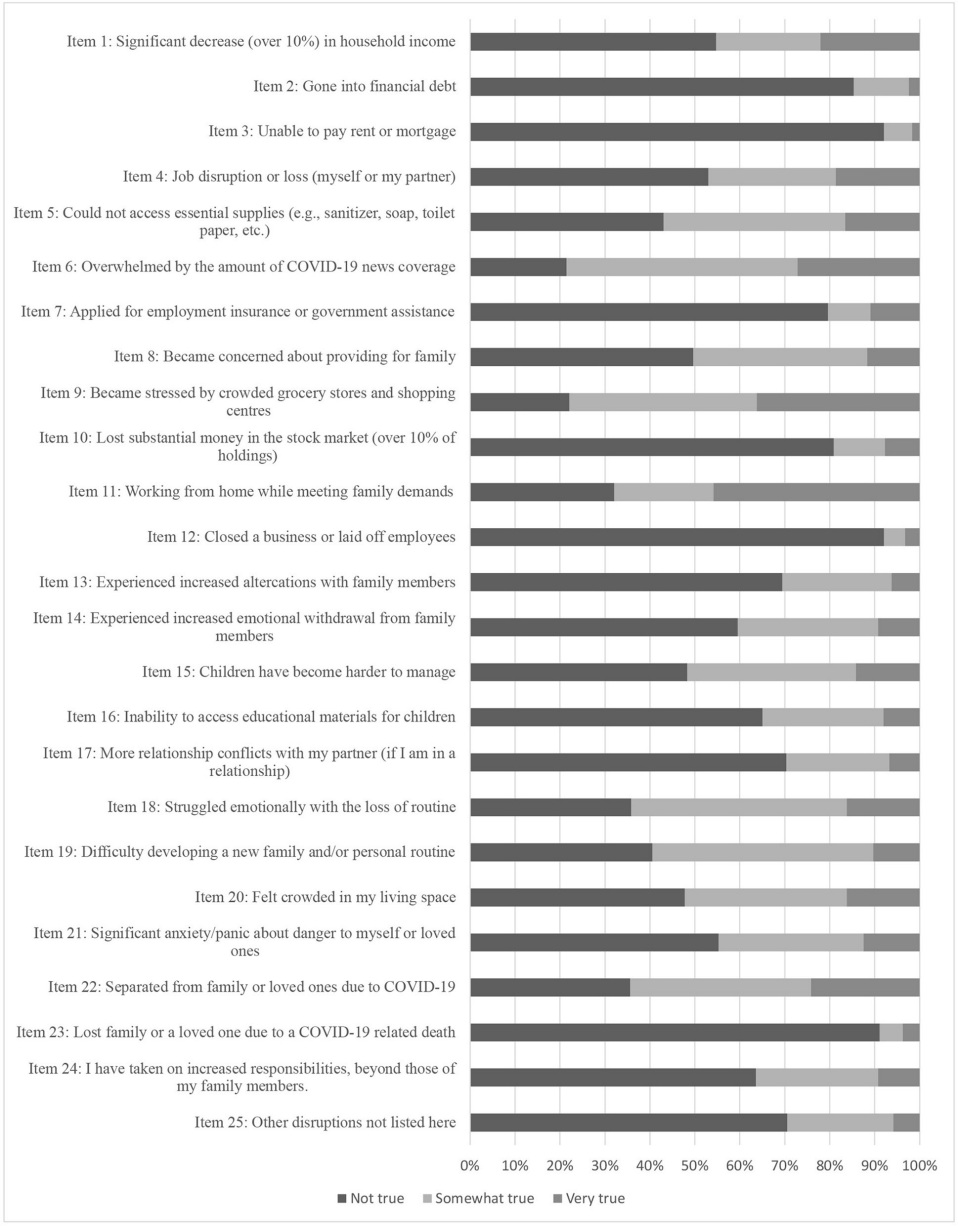
Distribution of responses on individual scale items of the COVID-19 Family Stressor Scale.

Distributions of item responses for the 25 scale items can be seen in the stacked bar plots in Figure 1. Three of the original 25 scale items were dropped due to low frequency endorsements (“Lost family or loved one due to COVID-19 related death;” “Closed a business or laid off employees;” “Unable to pay rent or mortgage”). The remaining 22 items were subjected to an EFA. A three-factor model was selected, representing a conceptually-coherent and meaningful factor solution, with good fit (CFI = 0.959; RMSEA = 0.047). Factor loadings and items can be seen in Table 2. The content of the items comprising the three factors were meaningfully interpretable as those reflecting stress due to: (i) income; (ii) family; and (iii) chaos related to COVID-19. These were therefore named Income Stress, Family Stress, and Chaos Stress, respectively. Five items did not meaningfully load onto the three-factor solution and were dropped from subsequent analyses (items 10, 11, 22, 24, 25). Finally, two items were found to be redundant (i.e., items 18 and 19), as is reflected by the wording of the items related to managing routines as well as high inter-correlations (*r* = 0.60 in the female caregiver group). Item 19 was retained for parsimony (due to its slightly higher factor loading). The final three factor solution included Income Stress (5 items with geomin rotation loadings ranging from 0.57 to 0.91); Family Stress (seven items ranging from 0.57 to 0.87); and Chaos Stress (four items ranging from 0.53 to 0.70). Items included in each of the three factors are denoted in Table 1. The three factors were significantly correlated in the EFA (ranging from 0.34 to 0.58, *p* < 0.05).

**Table 2:** Exploratory factor analysis three-factor solution (geomin rotated loadings).

**Item #**	**Item description**	**1**	**2**	**3**
1	Significant decrease (over 10%) in household income	0.91^*^	−0.00	−0.24
2	Gone into financial debt	0.65^*^	0.03	0.12
4	Job disruption or loss (myself or my partner)	0.79^*^	0.03	−0.11
5	Could not access essential supplies (e.g., sanitizer, soap, toilet paper, etc.)	−0.01	0.02	0.57^*^
6	Overwhelmed by the amount of COVID-19 news coverage	−0.04	0.15	0.53^*^
7	Applied for employment insurance or government assistance	0.77^*^	−0.10	0.03
8	Became concerned about providing for family	0.57^*^	−0.02	0.36^*^
9	Became stressed by crowded grocery stores and shopping centers	0.03	0.04	0.70^*^
10	Lost substantial money in the stock market (over 10% of holdings)	0.07	0.33^*^	−0.19
11	Working from home while meeting family demands	−0.17^*^	0.16^*^	0.04
13	Experienced increased altercations with family members	−0.00	0.87^*^	-0.21^*^
14	Experienced increased emotional withdrawal from family members	0.01	0.68^*^	0.10
15	Children have become harder to manage	−0.08	0.79^*^	−0.01
16	Inability to access educational materials for children	0.11	0.57^*^	0.01
17	More relationship conflicts with my partner (if I am in a relationship)	−0.02	0.87^*^	−0.16
18	Struggled emotionally with the loss of routine	0.07	0.60^*^	0.21^*^
19	Difficulty developing a new family and/or personal routine	0.11	0.61^*^	0.16
20	Felt crowded in my living space	−0.03	0.66^*^	0.09
21	Significant anxiety/panic about danger to myself or loved ones	−0.00	0.20^*^	0.59^*^
22	Separated from family or loved ones due to COVID-19	−0.06	0.19^*^	0.26^*^
24	I have taken on increased responsibilities, beyond those of my family members.	0.08	0.26^*^	0.36^*^
25	Other disruptions not listed here	0.12	0.28^*^	0.28^*^

*Items retained in final factor solution are in bold*.

* *Significant at p < 0.05*.

### Measurement Invariance

Results from invariance models can be seen in Table 3. For each factor, separately, baseline measurement models were fit for each group of male (*n* = 158) and female (*n* = 372) caregivers. Fit statistics were acceptable for all factors in both male and female caregivers. In the Income Stress factor, freely estimated parameters of residual covariances were included for males (“Significant decrease [over 10%] in household income” and “Job disruption or loss [myself or my partner]”) and females (“Gone into financial debt” and “Concern about providing for family”), respectively, based on an examination of modification indices and theoretical justification. These two sex-specific estimates were included in subsequent invariance models involving the income factors.

**Table 3:** Structural and measurement invariance model fit indices.

	**X^**2**^ (df)**	**CFI**	**RMSEA**	**ΔCFI**	**ΔRMSEA**	**ΔX^**2**^(df)**	**Decision**
**Three-factor EFA (full sample)**	**374.84 (168)**	**0.959**	**0.047**	**–**	**–**	**–**	**–**
**Measurement invariance: income stress factor**							
Female (*n* = 372)	5.10 (4)	0.999	0.027	–	–	–	–
Male (*n* = 158)	6.03 (4)	0.993	0.057	–	–	–	–
Configural invariance	11.10 (8)	0.997	0.038	–	–	–	–
Scalar Invariance^a^	21.29 (16)	0.995	0.035	−0.002	−0.003	10.51 (8)	Accept
**Measurement invariance: family stress factor**							
Female (*n* = 372)	32.95 (14)^**^	0.984	0.060	–	–	–	–
Male (*n* = 158)	18.71 (14)	0.995	0.046	–	–	–	–
Configural invariance	51.50 (28)^**^	0.989	0.056	–	–	–	–
Scalar Invariance^a^	81.84 (40)^**^	0.980	0.063	−0.009	0.007	30.26 (12)^**^	Accept
**Measurement invariance: chaos stress factor**							
Female (*n* = 372)	2.92 (2)	0.997	0.035	–	–	–	–
Male (*n* = 158)	4.81 (2)	0.984	0.094	–	–	–	–
Configural invariance	7.72 (4)	0.993	0.059	–	–	–	–
Scalar Invariance^a^	12.39 (10)	0.995	0.030	0.002	−0.029	5.54 (6)	Accept

** *< 0.005*;

a *Compared to Configural; CFI, comparative fit index; RMSEA, the root mean square error of approximation; X^2^, chi-square*.

*X^2^, CFI, RMSEA are absolute model fit indices, whereas* Δ*CFI, *Δ*RMSEA, and* Δ*X^2^ compare two nested models*.

#### Configural Model

The least restrictive model of configural invariance, without any constraints, fit the data well for each of the three factors, indicating that the factor structure was invariant across male and female caregivers. There was one exception for the Chaos Stress factor in males only, with one of the two fit statistics above the cut-off (RMSEA = 0.094). An examination of the probability of the RMSEA being ≤ 0.05 indicated a value of 0.179, indicating acceptable fit (40).

#### Scalar Model

Model fit for scalar invariance, wherein all factor loadings and item thresholds were constrained to equivalence across groups, was also acceptable across factors, and was not significantly worse than the configural invariance models for any of the factors. For the Family Stress factor only, the Chi-Square Difference Test [Scalar vs. Configural] was 30.26, *p* < 0.005, suggesting worsened fit. However, the less sensitive cut-offs of ΔCFI and ΔRMSEA did not indicate meaningful change in model fit (−0.009 and 0.007, respectively) (24, 39). Thus, we conclude that there is evidence for configural, metric, and scalar invariance in factors related to Income Stress, Family Stress, and Chaos Stress, respectively, across male and female caregivers.

### Scale Formation and Concurrent Validity

Composite variables based on summing of items were computed based on the final factor solution of the EFA. Each scale demonstrated acceptable internal consistency based on Cronbach's Alpha: Income Stress (five items, α = 0.75), Family Stress (seven items, α = 0.82), and Chaos Stress (four items; α = 0.68). Spearman's rho correlations indicated significant associations among constructed CoFaSS composites: Income Stress significantly correlated with Family Stress (*r*_*s*_ = 0.27, *p* < 0.001) and Chaos Stress (*r*_*s*_ = 0.33, *p* < 0.001). Family Stress and Chaos Stress had a large association (*r*_*s*_ = 0.50, *p* < 0.001). Given the significant intercorrelations between factors reported in the EFA, as well as between composite scores, a General Stress scale was justified and computed (16 items; α = 0.83).

Mann-Whitney *U* tests indicated that female caregivers scored higher than male caregivers on the General Stress scale, *z* = −5.18, *p* < 0.001 (difference in mean ranks = 75.20). Female caregivers also scored higher than male caregivers on the Income Stress subscale, *z* = −3.09, *p* < 0.001 (difference in mean ranks = 44.12), the Family Stress subscale, *z* = −3.94, *p* < 0.001 (difference in mean ranks = 56.88), and the Chaos subscale, *z* = −5.10, *p* < 0.001 (difference in mean ranks = 73.43).

The General Stress scale and each of the Income Stress, Family Stress, and Chaos Stress factors were correlated with constructs expected to relate to social disruptions from COVID-19, including caregiver (i.e., depressive symptoms, anxiety, parenting stress), family (i.e., marital satisfaction and conflict, parenting practices) and child outcomes (i.e., anxiety, depressive symptoms, anger; Table 4). Spearman rho correlations between the CoFaSS scales and caregiver outcomes were in the small to large range. Correlations between the CoFaSS scales and family and child outcomes, respectively, were in the small to medium range. The Family Stress subscale, as compared to the other subscales, was most consistently related to caregiver, family, and child outcomes (in the medium to large range), whereas the Income Stress subscale yielded the smallest associations with all outcomes (in the non-significant to small range), as compared to the other subscales.

**Table 4:** Spearman rho correlations between the CoFaSS scales and caregiver, family, and child outcomes.

	**CoFaSS scale**			
	**General**	**Income**	**Family**	**Chaos**
	**stress**	**stress**	**stress**	**stress**
**Caregiver outcomes**				
Anxiety	0.50^**^	0.22^**^	0.44^**^	0.47^**^
Depressive symptoms (*n* = 527)	0.54^**^	0.23^**^	0.52^**^	0.46^**^
Parenting stress (*n* = 530)	0.31^**^	0.04	0.42^**^	0.19^**^
**Family outcomes**				
Couple satisfaction^a^ (*n* = 488)	−0.27^**^	−0.11^*^	−0.35^**^	−0.07
Marital conflict^a^ (*n* = 488)	0.34^**^	0.12^**^	0.41^**^	0.17^**^
Parenting practices (*n* = 530)	−0.20^**^	−0.03	−0.29^**^	−0.07*^†^*
**Child outcomes**				
Depressive symptoms (*n* = 530)	0.29^**^	0.10^*^	0.36^**^	0.19^**^
Anxiety symptoms (*n* = 530)	0.33^**^	0.19^**^	0.30^**^	0.26^**^
Anger (*n* = 530)	0.27^**^	0.11^*^	0.31^**^	0.17^**^

*n, sample size included in analysis*;

** *Correlation is significant at the 0.01 level*;

* *Correlation is significant at the 0.05 level*;

† *Correlation is marginally significant at the 0.10 level*.

a *Valid missingness due to skips (no partner)*.

## Discussion

Assessing the extent to which families' lives are disrupted by the COVID-19 pandemic is critical to informing population-level policies as well as identifying vulnerable families in need of targeted services. Efforts to delineate the pathways through which the pandemic is adversely impacting family relationships, interactions, and mental health of family members relies on psychometrically sound measurement of stressors related to COVID-19. Without such measurement, it is not possible to quantify the individual and family differences in exposure to social disruptions related to this global health crisis. This study investigated the psychometric properties of a measure of family-related stressors emanating from the COVID-19 pandemic for female and male caregivers. A three-factor solution emerged, with factors reflecting stressors related to Income Stress, Family Stress, and Chaos Stress. Various sources of evidence support the construct validity of the CoFaSS, including the internal structure and measurement invariance of the individual factors across male and female caregivers, adequate internal consistency of the subscales, and significant associations with outcomes expected to vary by stress exposure.

Clustering of scale items represented three sources of stress to families during the pandemic. The first source of stress comes from income-related concerns (i.e., Income Stress subscale), including income reduction, debt, and job insecurity. Family-related stressors include those stemming from an increase in family altercations, emotional withdrawal, and child management concerns (i.e., Family Stress subscale). A third cluster of concerns emerged related to chaotic states such as difficulties accessing essential supplies and/or exposure to COVID-19 news coverage (i.e., Chaos Stress subscale). Thus, a particular clustering of stress may occur within individual families, with some strained due to financial insecurity, others due to the exacerbation of family issues arising from stay-at-home orders and social distancing measures, and still others that are due to disequilibrium of living during a time of severe public health threat. Differences in the sources of stress may have important implications for how families are impacted, pathways to resilience, and/or methods of intervention. Furthermore, the Income, Family, and Chaos Stress factors were significantly inter-correlated, suggesting that there may be an elevated climate of stress within families that stems from all three domains. In other words, these manifold stressors may aggregate together in some families, creating a particularly elevated threat to healthy adjustment and coping during the pandemic. Importantly, we might expect for the three types of COVID-19 stress to more frequently cluster in the most socially disadvantaged families, as has been demonstrated in studies of cumulative risk (41). It will be important to capture the ways in which pre-existing vulnerabilities exacerbate the impact of COVID-19 stress and disruption.

The emergence of three stress-related factors has important methodological implications for future family research. Specifically, the Family Stress factor indexes strain in family relationships and problems with child behavior management. This was reflected in the concurrent validity assessment wherein this subscale had the strongest associations with family and child outcomes among the three subscales. Future investigators can therefore decide when to use the General Stress scale or specific subscales based on specific study questions. For example, an investigation on the impact of pandemic-related stress on family processes may be better suited to using the Chaos and/or Income Stress subscales, so as to not conflate family stress with family process. As was demonstrated, use of the General Stress scale and/or specific subscales is a valid approach based on demonstration of reliability and concurrent validity, and should be tailored to specific samples, study designs, and research questions.

Validation of the CoFaSS in both male and female caregivers was a primary goal of the current study. Configural, metric, and scalar invariance of factors provides evidence that the meaning of the CoFaSS scale is consistent across male and female caregivers. That is, the structure (i.e., pattern of loadings) and contribution of each item to the factor (i.e., factor loadings) were similar across male and female caregivers, and the mean differences in the factors captured all mean differences in the shared variance of the items. Such a demonstration—referred to as strong measurement invariance—is required prior to using a measure for sex-based analysis (e.g., examining mean differences and/or interpreting regression coefficients, either across the entire sample or in a multi-group analysis) (24). This represents an important step for future investigations into the differential impact of the pandemic on male and female caregivers. Our hope is that the demonstrated validity of the CoFaSS in both groups of caregivers will facilitate research examining the disparate impact of social disruptions related to COVID-19 on male and female caregivers, and the downstream effects on whole families and children.

### Sampling and Generalizability

The validation of the CoFaSS and generalizability of findings should be interpreted in light of the sampling approach used in the current study. The majority of participants identified themselves as White-European. As such, these findings are not generalizable to diverse ethnic groups and racialized communities who are disproportionally impacted by global crises including COVID-19 (17, 42). Relatedly, the median household income in 2019 of the current sample fell in the $50,000 to $75,000 USD range, which is in line with the median household income in the United States ($68,703 USD) (43) and above that of the United Kingdom ($40,848 USD) (44). Furthermore, the majority of participants reported being in married/common-law relationships and had at least some post-secondary education. Subsequent use of the scale may benefit from targeted sampling approaches to allow for an examination of the validity and measurement equivalence across diverse ethnic/racial and socioeconomic groups. For example, correlations may be stronger between COVID-19-related stress and caregiver, family, and child outcomes in samples of individuals with pre-existing risk factors such as socioeconomic hardship, a history of developmental/mental health concerns, and/or experiences of marginalization (20).

Findings also need to be interpretated within the regional and time-related parameters of the study. Data for the current study were from May 2020, relatively early on in the pandemic. As such, items that were removed due to low frequency of endorsements at this timepoint may indeed be relevant at different times of the pandemic. Relatedly, a large proportion of the sample was from the United Kingdom and the United States, with smaller numbers from Canada and Australia. Different countries, and regions within countries, varied in the timing and magnitude of the COVID-19 threat (e.g., infection and death rates), as well as resulting policies (e.g., lockdown measures, income supplements). Limitations of our data, including measurement of the CoFaSS at a single time point, as well as small sample sizes within countries, preclude our ability to examine variations in stress over time or across regions. Given the ever-changing nature of the pandemic, it is important to consider the changing nature of family stressors. This is an issue that will be examined with subsequent data collection in the CRAMPED study.

Finally, data were collected as part of a larger within-family study requiring participants to have two or more children between the ages of 5–18 years in the household. Further validation efforts will be needed to extend the use of the CoFaSS to caregivers of younger children and/or those with only one child as the experiences may not generalize to these groups. For instance, there is some indication for protective effects of having siblings in the home in the prediction of children's well-being during COVID-19 (45, 46).

### Limitations

There are a few additional limitations that should be considered. First, caregivers reported on their own COVID-19-related stressors, caregiver, family, and child outcomes, thus raising the possibility of inflated associations due to shared-informant biases. Future validation procedures will be strengthened through a multi-informant and/or multi-method approach to address this threat to internal validity. Second, the readability of the scale items, as assessed by the Flesch-Kincaid Readability analysis, was a grade level of 9.6. This may limit comprehension amongst a broad range of groups, though at present is not considered prohibitive. Third, regarding validation procedures, additional sources of evidence for validity (e.g., cognitive processes during item responding) were beyond the scope of the paper and should be considered in future validation efforts (47). Finally, the current study only includes measurement on caregiver-reported sex, with “male,” “female,” and “prefer not to answer” options provided by Prolific®, and does not capture the complexity of gender expression (e.g., men, women, gender diverse people). As sex and gender do not always correlate, a two-step method, wherein participants are asked to identify their biological sex as indicated on their original birth certificate as well as their current gender identity, would have been a more comprehensive approach (48, 49).

## Data Availability Statement

The raw data supporting the conclusions of this article will be made available by the authors, without undue reservation.

## Ethics Statement

The studies involving human participants were reviewed and approved by The Human Research Ethics Committee–University of Waterloo; The Human Participants Review Subcommittee–York University Ethics Review Board; Research Oversight and Compliance, University of Toronto. Participants provided electronic informed consent by selecting “Yes” on the following item: “Do you agree of you own free will to participate in this study?” If participants selected “No”, they were routed to an ineligibility message and were unable to participate.

## Author Contributions

HP: conceptualization, analysis and writing. MW and JJ: reviewing and editing. SM: data collection and management and reviewing and editing. DB: conceptualization, methodology, data collection, and reviewing and editing. All authors contributed to the article and approved the submitted version.

## Conflict of Interest

The authors declare that the research was conducted in the absence of any commercial or financial relationships that could be construed as a potential conflict of interest.
